# Knowledge, Attitude, and Practice (KAP) Survey toward Skin Cancer among Ecuadorian Population

**DOI:** 10.1155/2021/5539149

**Published:** 2021-08-04

**Authors:** Victor H. Pinos-León, César Sandoval, Franklin Cabrera, Esmeraldas Terán, Ana Garnica, Ana Kellendonk, Mery Alvear, Carla Rosero, Lorena Vaca, Jorge Bonifaz, Anita Buestán, Claudia Armas, Raquel Trujillo, Patricio Freire, Tamara León, Giselle Erazo, Liliana García, Marcela Alzate, Vicente Toapanta, Myriam Ortega, Daniela Caicedo, Alicia Pereira, Lenin Gómez-Barreno, Juan S. Izquierdo-Condoy, Merry Charlie, Esteban Ortiz-Prado, Katherine Simbaña-Rivera

**Affiliations:** ^1^Dermatology Department, Hospital Metropolitano de Quito, Quito 170137, Ecuador; ^2^Dermatology Program Faculty of Medicine, Universidad Central del Ecuador, Quito 170118, Ecuador; ^3^Dermatology Department, Hospital Carlos Andrade Marin del IESS, Quito 170118, Ecuador; ^4^Staff Physician Centro Quirúrgico Láser Visión y Piel, Quito 170135, Ecuador; ^5^Dermatology Department, IESS Comité del Pueblo, Quito 170133, Ecuador; ^6^Dermatology Department, Hospital Padre Carolo, Quito 170146, Ecuador; ^7^Dermatology Department, Hospital General Docente de Calderón, Quito 170155, Ecuador; ^8^Dermatology Department, Hospiral Enrique Garcés, Quito 170135, Ecuador; ^9^Dermatology Department, Hospital Quito No. 1 Policía Nacional, Quito 170137, Ecuador; ^10^Ecuadorian Society of Dermatology, Quito 170105, Ecuador; ^11^Dermatology Program Faculty of Medicine, UTE, Quito 170137, Ecuador; ^12^One Health Research Group, Universidad de las Américas, Quito 170513, Ecuador; ^13^University of Southampton, Southampton SO16 7PP, UK; ^14^Latin American Network for Cancer Research (LAN-CANCER), Lima, Peru

## Abstract

**Background:**

Skin cancer is one of the most common cancers, and melanoma is a highly preventable cancer. In Ecuador, few studies have evaluated the awareness levels of the population about the disease. For this reason, the objective of this study was to measure the level of knowledge, attitudes, and practices regarding skin cancer and its determining factors.

**Methods:**

A cross-sectional analysis using an online self-assessment questionnaire containing 40 questions was delivered. A total of 537 participants were included in this study. Knowledge, attitude, and practice scores were assigned to each participant based on the number of correct or appropriate responses. Logistic regression analysis was used to calculate crude and adjusted odds ratios.

**Results:**

In total, 75% of participants referenced knowledge of the harmful effects related to noncontrolled solar exposure. Concerning sunscreen, 76.7% knew the reason for using it. The female group was 1.68 times more likely to get a higher score than the male group, and the groups between 61–70 and 71–80 years were 0.30 and 0.17 times less likely to get a higher score compared with the less than 20-years-old group, respectively.

**Conclusions:**

The findings of this study indicate the requirement to increase the population's knowledge about skin cancer and possible protection measures. For this reason, the prevention and health promotion programs at a national level from primary healthcare centers are recommended. Due to the limitation of the representativeness of the sample, the use of more studies among Ecuadorian residents of the low socioeconomic level and replication in different provinces of Ecuador is justified.

## 1. Introduction

Cancer is the second leading cause of death worldwide, with skin cancer as one of the most common. Melanoma is the most clinically relevant skin cancer, either in frequency or mortality [1, 2], whose trend has increased over the years [3, 4].

Diagnosis increases after 60 years of age. It is 1.5 times more frequent in men than in women, and this may be due to the fact that they are more exposed to UV radiation due to economic and work activities. Similarly, the incidence is higher in people with fair skin [5].

The risk of developing skin cancer depends on constitutional factors such as skin phototypes I and II, solar lentigines, dysplastic nevi, immunosuppression, a family history of melanoma [6, 7], and environmental factors such as altitude, latitude, and season of the year.

Excessive exposure to ultraviolet (UV) radiation can be considered the main cause, especially in recent years due to the reduction in the ozone layer. It has shown significant differences between UVA and UVB radiation, such as ionizing radiation and the ability to penetrate the skin, with UVB being the main cause of cutaneous neoplasms, even though it only represents 5% of solar radiation. This is because UVB radiation causes the appearance of point mutations in target genes [8–10].

According to the “Sociedad de Lucha contra el Cáncer” (SOLCA) data, cutaneous neoplasms are the third most frequent cancer in the country, and Quito presents the highest number of cases [11]. This could be due to the fact that the capital is 2850 meters above sea level; in the equatorial zone, the solar rays fall in a direct way so that in the middle latitudes and the UV radiation are consequently more intense in this area. In addition, as Quito is located in a high mountainous area, it facilitates the path of solar rays; so that at higher altitudes, there is greater UV radiation [12].

The increased incidence of skin cancer is due to diagnostic deficiencies in both primary and secondary care, as well as unnecessary exposure to environmental factors by the population, in many cases due to lack of knowledge on the subject [8, 13, 14].

In Ecuador, few studies have systematically evaluated the level of awareness of the population about the disease. For this reason, the objective of the present study was to measure the level of knowledge and the attitudes and practices regarding skin cancer and its determining factors.

## 2. Materials and Methods

### 2.1. Study Setting

The population of Ecuador is estimated at 17.3 million [15]. This study was conducted in three different zones of Quito—North, Center, and South—with a total population of 3.1 million (18% of the entire country).

### 2.2. Study Design

A cross-sectional study of the primary sources was carried out during the dissemination of the campaign of prevention and health promotion for the “Melanoma Day” organized by the Ecuadorian Society of Dermatology in Quito, Ecuador, in May 2019.

### 2.3. Study Size and Participants

Participants were between 15 and 80 years old. The aim of the study was explained to potential participants, who then agreed to participate in this study and signed the informed consent form. Participation was strictly voluntary, and no incentives were provided.

The sample size was calculated with a 95% confidence level, obtaining a minimum of 385 subjects. The real sample size was 537 respondents.

In addition, all participants who rejected informed consent and provided incomplete questionnaires (*n* = 56) were excluded from the study.

### 2.4. Study Instrument and Data Collection

A survey previously reviewed and validated by experts was applied after a pilot study with ten people from different age groups. Five difficult-to-understand questions were identified and reformulated. In addition, the interviewers were instructed on the application of the survey in general and the specifications to be given to the participants in those questions. The questionnaire was composed of a few sections: sociodemographic characteristics (sex, age, marital status, education, occupation, income, number in household), knowledge, attitudes, and practices about the sunscreen use to prevent skin cancer.

### 2.5. Data Analysis

Characteristics of the study population were analyzed using descriptive statistics (frequency and percentage). Knowledge, attitude, and practice scores were assigned to each participant based on the number of correct or appropriate responses. An appropriate answer was assigned one point. The knowledge and attitude scales were 0–12 points, and the practice scale was 0–20 points. Indeed, the scores were dichotomized into low (poor) or high (good) taking into account the mean of each category as follows: knowledge (low = 0–5 points; high = 6–12 points); attitude (poor = 0–5 points; good = 6–12 points); and practice (poor = 0–11 points, good = 12–20 points). Logistic regression analysis was used to calculate crude and adjusted odds ratios as well as corresponding 95% confidence intervals (CI). Each variable was first entered separately into bivariate logistic regression models to evaluate the crude association with sociodemographic characteristics. All predictors were then entered in multivariable logistic regression to get adjusted odds ratios (ORa). Data were analyzed with SPSS version 21 software.

## 3. Results

### 3.1. Sociodemographic Characteristics

There were a total of 537 participants in this study. Of these, 60.5% (*n* = 325) were female. The median age of the study participants was 41.5 years (IQR: 19–58). The most common age subgroup was between 51 and 60 years. Concerning race, 92.9% (*n* = 499) of the participants identified themselves as mestizo followed by 4.1% (*n* = 22) as indigenous. Regarding marital status, 54.4% (*n* = 292) were married and 26.8% (*n* = 144) were single (Table 1). Relating to nationality, only 1.9% (*n* = 10) were non-Ecuadorian. According to the demographic factors of the participants, 95.7% were residents of the Quito city.

The most relevant social factor was that family income for 43.2% (*n* = 232) corresponded to the interval between 1 and 2 unified minimum wages followed by 30.2% (*n* = 162) who earned less than 1 minimum wage. According to the number of inhabitants per house, 60% (*n* = 322) answered that they shared with between 2 and 4 people. Within the educational level, it was observed that 43.4% (*n* = 233), 29.2% (*n* = 157), and 19.6% (*n* = 105) had completed the secondary, primary, and third level of education, respectively.

The majority of the participants reported that they wrought as housewives 25.7% (*n* = 138) followed by professionals 17.5% (*n* = 94), students 15.6% (*n* = 84), among others. In addition to working conditions, 60.7% (*n* = 326) reported working in urban areas, while suburban and rural levels counted for only 6.9% (*n* = 37), and the rest were not working at the time of the survey. On the other hand, the residential conditions of the registered participants demonstrate that 91.8% of the participants live in urban areas (*n* = 493), while the rest live in suburban or rural areas. According to the basic services, it is evident that 1.1% (*n* = 6), 1.3% (*n* = 7), and 0.2% (*n* = 1) had no drinking water, sewage, and electricity services, respectively (Table 1).

Of the respondents, only 1.7% (*n* = 9) had a personal history of skin malignancy, and 6.1% reported having a family history of it. On the other hand, almost half of the participants (*n* = 225; 41.9%) reported having type IV skin according to the Fitzpatrick classification (Table 1).

### 3.2. Knowledge

Approximately 75% of participants (*n* = 400) referenced knowledge of the harmful effects related to noncontrolled solar exposure. Of those participants, 49.3% knew several effects; 22% linked it to skin tumors followed by 9.9% to solar burns, 6.5% to skin spots, and others (Table 2). Concerning causality, 72.3% (*n* = 388) of participants agreed that there is a direct relationship between noncontrolled solar exposure and skin cancer. Concerning sunscreen, most people, 74.5% (*n* = 400), answered that they knew of the concept and types, and 76.7% knew the reason for using it. Finally, 62.2% of the participants reported knowing other protection measures in addition to the use of sunscreen.

Concerning the regression results, in the bivariate analysis, it was demonstrated that people with a monthly income higher than $1000 were 2.36 times more likely to score higher on the knowledge scale compared with those with an income lower than a minimum living wage. Related to education, people who have finished the secondary, technician, tertiary, and fourth level were 9.92, 20, 23.87, and 12.5 times more likely to score higher compared with people with no education, respectively. In addition, relating to employment, the categories of housewife, trade, students, and unemployment were 0.44, 0.57, 0.5, and 0.23 less likely to score higher compared with professionals, respectively. However, after appropriate adjustments were made, all study levels remained unaffected, while for other categories only students were still less likely 0.24 (0.07–0.91) to have higher scores compared with professionals.

### 3.3. Attitude

Approximately half of the participants (*n* = 269; 50.1%) reported having presented more than three burns caused by solar radiation during their lifetime. Regarding the amount of exposure to solar radiation during childhood and adolescence, the majority of participants, 71.3% (*n* = 383), reported having been exposed without protection for more than 6 days in each week during their development.

About sun protection factor (SPF) present in sunscreens, the most commonly used were more than 50% described by 24.6% (*n* = 172) followed by the range of 30 to 50% described by 16.6% (*n* = 89). A third of respondents, 37.4% (*n* = 201), reported that the approximate value of a sunscreen is in the range of 11 to 20 US dollars. In addition, 61.9% of the participants (*n* = 332) reported that this cost is affordable. Finally, half of these products are purchased in pharmacies, 48.8% (*n* = 262), while the rest of the products were purchased in stores, catalogs, among others (Table 3).

In bivariate analysis, the female group was 1.63 times more likely to score higher on attitude score than the male group. According to marital status, it was observed that people who live with a partner (married/consensual union) were 0.62 times less likely to score higher compared with those with no partner (single/divorced/widowed). Another important finding was that the age groups 61–70 and 71–80 both presented less likely to score higher compared with those <20 years. Moreover, for monthly income, it was evidenced that both groups ($650–$1000 and more than $1000) had 1.95 and 3.72, respectively, more likely to obtain higher scores compared with the less-income group. Concerning education, only secondary, tertiary, and fourth level were 9.92, 16.92, and 20 times more likely to get higher scores compared with the noneducated group, respectively. Finally, compared with professionals, trades and retired were 0.43 and 0.31 times less likely to score higher, respectively. In contrast, when multivariate analysis was performed. Adjusted results that remains significant were live with a partner (ORa = 0.57), income over $1000 (ORa = 2.73) and secondary, tertiary and fourth level of education with (ORa = 9.9), (ORa = 16.47), and (ORa = 18.95), respectively (Table 4).

### 3.4. Practice

Close to half of the participants reported daily sun exposure of less than two hours in 42.1% (*n* = 226) and three to five hours in 39.5% (*n* = 212). Regarding protection practices, 70.4% (*n* = 378) of participants had some type of sunscreen at home. Furthermore, 63.1% (*n* = 339) use it regularly. Of this group, approximately 50.4% (*n* = 270) use it every day, while the other half only use it occasionally or on weekends. Furthermore, 71.5% (*n* = 384) reported that they use it once a day, and only 6.4% (*n* = 34) use it three times a day. Likewise, 55.3% (*n* = 297) reported using it all year round, while the rest used it according to the season, most 43.7% (*n* = 234) in summer (June–November) (Table 5).

The results of bivariate analysis demonstrated that the female group is 1.68 times more likely to get a higher score than the male group. In addition, related to age, it was observed that the groups between 61–70 and 71–80 years were 0.30 and 0.17 times less likely to get a higher score in the practice section compared with the <20-years-old group. Concerning the level of education, the primary, secondary, technician, tertiary, and fourth level groups were 9.38, 18.77, 15, 23.87, and 80 times more likely to get higher values on practice scores compared with no education group, respectively. Moreover, it was found that trade and retired groups were 0.37 and 0.27 less likely to get a higher score compared with professionals, respectively. Lastly, it was shown that the group that lives outside the city was 0.48 times less likely to get a higher score compared with urban residents. However, after appropriate adjusted comparison, there still remained differences per sex (ORa = 1.71); per age in the group 61–70 (ORa = 0.16) and group 71–80 (ORa = 0.15); per family income over $1000 (ORa = 3.97); per education in secondary, technician, tertiary, and fourth levels (ORa = 16.51, 11.51, 17.57, and 62.18, respectively); and per residential location (ORa = 0.41) (Table 4). Suddenly concerning the household, it was shown that the group from 2 to 4 people in the house which increases 2.17 times more likely to obtain a higher score compared with less than 2 persons. Finally, another important fact is that only the student group was 0.24 times less likely to get higher practice scores compared with professionals.

## 4. Discussion

Skin cancer is currently among the most common cancers, showing increasing incidence and mortality rates [16, 17]. In Ecuador, skin cancer is found in the three most frequent SOLCA malignancies. This study was carried out to evaluate the level of knowledge and the attitudes and practices regarding skin cancer and sunscreen in the adult population of the city of Quito. The data obtained concerning the level of knowledge showed a direct association between socioeconomic level and its indicators, such as monthly income greater than $ 1000, as well as those with a higher academic degree, results that are consistent with those presented in the studies of Rhazi et al. [18]. This was also found associated with the workplace and residence, with the urban population achieving the highest levels of knowledge, compared with the rural population, a relationship that has also been evidenced in a study by Rhazi et al. in Moroccan population [18].

Sun exposure during childhood has been recognized as a risk factor for the development of skin cancer in adult life [19]. In this context, 49.2% of the respondents have presented at least three sunburns during their childhood.

The phototype is a risk factor for the development of skin cancer. Studies carried out in populations located in eastern regions show a lower prevalence of skin cancer than in Western countries [20]. However, probably due to the geographical location of Ecuador, the most frequent phototypes in our study, according to the Fitzpatrick scale, were type IV in 42% of the participants and type III in 30%, Mufti et al. [21] showed that low skin cancer rates in dark phototypes in the Arab population, and the research by Yan et al. [20] carried out in Chinese population showed that most of them present a type IV skin phototype with a low prevalence of skin cancer. Correspondingly, only 1.7% of our respondents have a personal history of skin malignancies and only 6.1% had a family history.

Compared with studies carried out in defined populations, we found that knowledge about the general risk of exposure to sunlight was present in 75% of participants, among them 72% reported knowing the existence of a relationship between sun exposure and the development of skin cancer. However, in a study of 387 Greek students, skin cancer (97.7%) was identified as the main consequence followed by skin aging in 72.9%; similar results have been shown in Arab and United States students [22–24].

Concerning sex, men have shown less interest in skin care compared with women due to cultural gender roles [25]. This is supported by the data of Almuqati et al. [23], where women present more knowledge about the consequences of sun exposure including burns, hyperpigmentation, aging, and skin cancer. Despite not finding differences on the level of knowledge regarding sex in our study, it was evidenced that female participants were more likely to develop attitudes (OR: 1.63; CI: 1.15–2.31) and practices (ORa: 1.71; CI: 1.04–2.80) for skin care and protection against sun rays and skin cancer.

About the sources of information dissemination, the perception of the participants of our study showed 69% as the main source of information as a favorite of the media, television, radio, and shops, a behavior seen in a similar way in the study of Wan [19] carried out in China's adult population that chose television ads as the main source of information (52%) on sun protection. However, only 8% of our participants took data from professional sources such as doctors, dermatologists, and health campaigns.

A variety of protective measures have been described to reduce the incidence of skin cancer, at the moment the best measures are to limit exposure to sunlight, especially during peak hours and the use of sunscreen [26, 27]. Unlike the data provided by Almuqati et al. [23], which showed that only 23.6% of Arab students use sunscreen regularly, while 63% of the population in our study does, this difference was possibly influenced by the fact that different age groups of adults make up the population and not only students [23]. In addition, 45% erroneously maintain that the use of sunscreen is not necessary on cloudy days with or without sun, a belief also accepted by Mexican inhabitants 47.1% [28].

Regarding the use of other sun protection measures, 70% of participants reported opting for alternatives to sunscreen, among these, the use of caps and hats were the most frequent in 53%. These measures are also carried out in Western populations from different regions, such as Mexico, where the use of sunglasses and caps as physical protection measures are present in 73% and 66.9%, respectively, of people who practice sports on the beach [28].

Due to the limitation in the representativeness of the sample, more studies are warranted to investigate the KAP toward skin cancer among Ecuadorians residents of low socioeconomic status and for subsequent studies it can be assessed in different provinces of Ecuador.

## 5. Conclusions

Skin cancer is one of the most frequent cancers in the country for both men and women; indeed it is a type of cancer that is highly preventable. The findings of this study indicate the need to increase the population's knowledge about skin cancer and the risk factors for its development, as well as possible protection measures. For this reason, the formulation of prevention and health promotion programs at a national level for primary healthcare centers is recommended.

## Figures and Tables

**Table 1:** Sociodemographic characteristics of respondents.

		Gender			
		Male		Female	
		(*n* = 212; 39.5%)		(*n* = 325; 60.5%)	
		*n*	%	*n*	%
Age groups	15–20 y/o	21	9.9	46	14.2
	21–30 y/o	18	8.5	35	10.8
	31–40 y/o	21	9.9	59	18.2
	41–50 y/o	35	16.5	73	22.5
	51–60 y/o	53	25.0	57	17.5
	61–70 y/o	45	21.2	44	13.5
	71–80 y/o	17	8.0	10	3.1
	>81 y/o	2	0.9	1	0.3

Ethnic self-identification	Mestizo	196	92.5	303	93.2
	Montubio	2	0.9	1	0.3
	Black	1	0.5	5	1.5
	Indigenous	7	3.3	15	4.6
	White	3	1.4	2	0.6
	Other	0	0.0	2	0.6

Marital status	Single	48	22.6	96	29.5
	Married	127	59.9	165	50.8
	Consensual union	7	3.3	33	10.2
	Separated	4	1.9	4	1.2
	Divorced	22	10.4	16	4.9
	Widowed	4	1.9	11	3.4

Years of residence	0–10	29	13.7	33	10.2
	11–20	52	24.5	96	29.5
	21–30	23	10.8	78	24.0
	31–40	32	15.1	49	15.1
	41–50	27	12.7	34	10.5
	51–60	24	11.3	18	5.5
	61–70	15	7.1	17	5.2
	>70	10	4.7	0	0.0

Family income ($)	<386	52	24.5	110	33.8
	386–650	97	45.8	135	41.5
	650–1000	47	22.2	46	14.2
	>1000	25	11.8	25	7.7

How many people live in your house?	<2 people	30	14.2	20	6.2
	2–4 people	125	59.0	197	60.6
	4–6 people	49	23.1	92	28.3
	>6 people	6	2.8	12	3.7

Level of education	Illiterate	4	1.9	7	2.2
	Preschool	2	0.9	5	1.5
	Elementary school	57	26.9	100	30.8
	Higher education	89	42.0	144	44.3
	Technical	9	4.2	6	1.8
	Superior third level	46	21.7	59	18.2
	Superior fourth level	5	2.4	4	1.2

Occupation	Worker	54	25.5	35	10.8
	Housewife	2	0.9	136	41.8
	Farmer	4	1.9	2	0.6
	Merchant	19	9.0	31	9.5
	Student	29	13.7	55	16.9
	Professional	62	29.2	32	9.8
	Military or police	1	0.5	1	0.3
	Retired	21	9.9	13	4.0
	Unemployed	12	5.7	12	3.7
	Mixed	8	3.8	8	2.5

Workplace	Inside	49	23.1	123	37.8
	Outside	75	35.4	36	11.1
	Both	29	13.7	37	11.4
	Not applicable	58	27.4	130	40.0

Workplace location	Urban	138	65.1	188	57.8
	Suburban	7	3.3	9	2.8
	Rural	12	5.7	9	2.8
	Not apply	57	26.9	117	36.0

Location of residence site	Urban	197	92.9	296	91.1
	Suburban	6	2.8	13	4.0
	Rural	9	4.2	15	4.6

Do you have drinking water?	Yes	210	99.1	321	98.8
	No	2	0.9	4	1.2

Do you have sewage services?	Yes	209	98.6	321	98.8
	No	3	1.4	4	1.2

Do you have electric lighting?	Yes	212	100.0	324	99.7
	No	0	0.0	1	0.3

Type of skin	Type 1	5	2.4	7	2.2
	Type 2	24	11.3	25	7.7
	Type 3	54	25.5	109	33.5
	Type 4	94	44.3	131	40.3
	Type 5	33	15.6	48	14.8
	Type 6	3	1.4	4	1.2

Personal history of skin cancer	Yes	3	1.4	6	1.8
	No	209	98.6	319	98.2

Family history of skin cancer	Yes	9	4.2	24	7.4
	No	204	96.2	300	92.3

**Table 2:** Knowledge questionnaire of skin cancer.

		Gender			
		Male		Female	
		(*n* = 212; 39.5%)		(*n* = 325; 60.5%)	
		*n*	%	*n*	%
Do you know the risks of sun exposure?	Yes	153	72.2	247	76.0
	No	59	27.8	78	24.0

What negative effects do you know?	Sunburn	27	12.7	26	8.0
	Wrinkles	0	0.0	4	1.2
	Aging	3	1.4	3	0.9
	Spots	15	7.1	20	6.2
	Freckles	3	1.4	6	1.8
	Dry skin	2	0.9	1	0.3
	Skin cancer	52	24.5	66	20.3
	None	17	8.0	27	8.3
	Multiple	93	43.9	172	52.9

Do you know about skin cancer?	Yes	98	46.2	150	46.2
	No	114	53.8	175	53.8

Is there a relationship between skin cancer and exposure to sunlight?	Yes	155	73.1	233	71.7
	No	57	26.9	92	28.3

Do you know what sunscreen is?	Yes	157	74.1	243	74.8
	No	55	25.9	82	25.2

Do you know what sunscreen is used for?	Yes	155	73.1	257	79.1
	No	57	26.9	68	20.9

Do you know of other sun protection measures apart from sunscreen?	Yes	127	59.9	207	63.7
	No	85	40.1	118	36.3

**Table 3:** Attitude questionnaires of skin cancer.

		Gender			
		Male		Female	
		(*n* = 212; 39.5%)		(*n* = 325; 60.5%)	
Do you consider the value accessible?	Yes	140	66.0	193	59.2
	No	72	34.0	132	40.8

What were your reasons not to use sunscreen?	Expensive	41	19.2	104	32.0
	Don't have time	8	3.8	7	2.0
	Forgot to put it	38	17.9	55	17.0
	Don't like the sensation	30	14.1	33	10.0
	I want a sun tan	0	0.0	0	0.0
	It's not necessary for my skin type	41	19.2	26	8.0
	It's not necessary all year round	8	3.8	7	2.0
	Others	22	10.3	46	14.0
	Multiple	24	11.5	49	15.0

Do you use any other different prevention measure to the sunscreen?	Yes	158	74.7	252	77.6
	No	54	25.3	73	22.4

Do you think people look healthier with a tan?	Yes	46	21.5	54	16.6
	No	166	78.5	271	83.4

When you go to the beach or a picnic, are you intentionally looking for a tan?	Yes	42	19.6	50	15.3
	No	170	80.4	275	84.7

On nonsunny, cloudy days, do you think it is necessary to use sunscreen?	Yes	100	47.1	196	60.3
	No	112	52.9	129	39.7

**Table 4:** Odds ratio (OR) and adjusted OR (ORa) for KAP.

Category	Knowledge		Attitude		Practice	
	OR	ORa	OR	ORa	OR	ORa
Sex						
Male vs. female	0.99 (0.70–1.41)	1.22 (0.76–1.96)	**1.63 (1.15–2.31)** ^*∗∗*^	1.59 (0.99–2.56)	**1.68 (1.18**–**3**–**40)**^*∗∗*^	**1.71 (1.04**–**2.80)**^*∗*^

Marital status						
In a relationship vs. single	0.71 (0.50–1.00)	0.62 (0.38–1.03)	**0.62 (0.43–0.87)** ^*∗∗*^	**0.57 (0.34–0.93)** ^*∗*^	0.76 (0.53–1.08)	0.61 (0.40–1.04)

Age (years)						
<20	Reference					
21 to 30	1.34 (0.65–2.77)	0.61 (0.19–2.00)	1.14 (0.55–2.37)	0.80 (0.25–2.56)	1.10 (0.49–2.48)	0.66 (0.20–2.24)
31 to 40	1.48 (0.77–2.84)	0.57 (0.13–2.54)	0.73 (0.38–1.41)	0.44 (0.10–1.91)	0.78 (0.38–1.57)	0.43 (0.01–2.06)
41 to 50	1.29 (0.70–2.37)	0.55 (0.13–2.34)	0.91 (0.49–1.67)	0.48 (0.12–2.00)	0.76 (0.39–1.48)	0.35 (0.08–1.6)
51 to 60	0.81 (0.44–1.49)	0.32 (0.08–1.40)	0.56 (0.30–1.04)	0.40 (0.09–1.67)	0.57 (0.30–1.10)	0.37 (0.09–1.69)
61 to 70	0.72 (0.38–1.36)	0.30 (0.07–1.34)	**0.41 (0.22–0.79)** ^*∗∗*^	0.30 (0.07–1.35)	**0.30 (0.15**–**0.58)**^*∗∗∗*^	**0.16 (0.03**–**0.74)**^*∗*^
71 to 80	0.79 (0.32–1.96)	0.49 (0.08–2.83)	**0.23 (0.08–0.65)** ^*∗∗*^	0.39 (0.07–2.37)	**0.17 (0.06**–**0.45)**^*∗∗∗*^	**0.15(0.02**–**0.92)**^*∗*^

Family income ($)						
<386	Reference					
386–650	1.35 (0.89–2.04)	1.12 (0.69–1.80)	1.47 (0.96–2.24)	1.30 (0.80–2.12)	1.48 (0.98–2.24)	1.29 (0.80–2.11)
650–1000	1.54 (0.92–2.58)	1.09 (0.59–2.01)	**1.95 (1.16–3.29)** ^*∗*^	1.45 (0.78–2.7)	1.69 (0.99–2.86)	1.47 (0.77–2.80)
More than 1000	**2.06 (1.07–3.97)** ^*∗*^	1.47 (0.67–3.25)	**3.72 (1.89–7.33)** ^*∗∗∗*^	**2.73 (1.21–6.17)** ^*∗*^	**5.12 (2.25**–**11.60)**^*∗∗∗*^	**3.97 (1.54**–**10.28)**^*∗∗*^

How many people live in the home?						
<2	Reference					
2 to 4	1.15 (0.23–5.77)	1.83 (0.90–3.73)	0.81 (0.27–2.47)	1.58 (0.76–3.30)	0.34 (0.04–2.96)	**2.17 (1.02**–**4.39)**^*∗*^
4 to 6	0.81 (0.16–4.14)	1.25 (0.58–2.71)	1.39 (0.53–3.67)	1.59 (0.72–3.53)	0.27 (0.03–2.37)	1.76 (0.80–3.90)
More than 6	1 (0.16–6.35)	1.71 (0.48–6.08)	1.38 (0.51–3.78)	0.97 (0.26–3.68)	0.31 (0.03–3.29)	2.18 (0.59–8.05)

Education						
Illiterate	Reference					
Preschool	7.5 (0.59–95.38)	9.05 (0.57–144.85)	1.67 (0.09–31.87)	2.41 (0.11–53.62)	4 (0.29–55.47)	7.31 (0.46–128.59)
Primary	6.02 (0.75–48.23)	6.27 (0.70–55.81)	4.27 (0.53–34.33)	5.71 (0.64–50.71)	**9.38 (1.17**–**75.05)**^*∗*^	12.92 (1.37–121.73)
High school	**9.92 (1.25–78.70)** ^*∗*^	**9.82 (1.10–87.79)** ^*∗*^	**9.92 (1.25–78.70)** ^*∗*^	**9.9 (1.12–87.39)** ^*∗*^	**18.77 (2.36**–**149.20)**^*∗∗*^	**16.51 (1.77**–**153.97)**^*∗*^
Technology	**20 (1.97–203–32)** ^*∗*^	**25.02 (2.07–302.16)** ^*∗*^	8.75 (0.88–86.60)	7.37 (0.65–84.04)	**15 (1.50**–**149.7)**^*∗*^	**11.51 (1.94**–**140.862)**^*∗∗*^
Third level education	**23.87 (2.93–194.53)** ^*∗∗*^	**24.20 (2.54–230–70)** ^*∗∗*^	**16.92 (2.09–137.28)** ^*∗∗*^	**16.47 (1.76–153.88)** ^*∗*^	**23.87 (2.93**–**194.54)**^*∗∗*^	**17.57(1.78**–**174.12)**^*∗∗*^
Fourth level education	**12.5 (1.09–143.43)** ^*∗*^	**14.3 (1.02–200.05)** ^*∗*^	**20 (1.68–238.63)** ^*∗*^	**18.95 (1.35–266.88)** ^*∗*^	**80 (4.30**–**1488.604)**^*∗∗*^	**62.18 (2.72**–**1219.40)**^*∗*^

Occupation						
Professionals	Reference					
Housewives	**0.44 (0.26–0.75)** ^*∗∗*^	0.79 (0.38–1.66)	0.73 (0.43–1.24)	1.31 (0.63–2.74)	0.69 (0.39–1.21)	1.01 (0.46–2.21)
Jobs	**0.57 (0.34–0.97)** ^*∗*^	1.19 (0.62–2.30)	**0.43 (0.25–0.72)** ^*∗∗*^	0.85 (0.45–1.64)	**0.37 (0.21**–**0.63)**^*∗∗∗*^	0.63 (0.32–1.23)
Students	**0.50 (0.27–0.91)** ^*∗*^	**0.24 (0.07–0.91)** ^*∗*^	0.86 (0.48–1.55)	0.33 (0.09–1.20)	0.87 (0.46–1.66)	**0.24 (0.06**–**0.95)**^*∗*^
Retirees	0.55 (0.25–1.21)	1.21 (0.44–3.35)	**0.31 (0.13–0.72)** ^*∗∗*^	0.67 (0.23–1–95)	**0.27 (0.12**–**0.62)**^*∗∗*^	0.72 (0.21–2.06)
Unemployed	**0.23 (0.09–0.60)** ^*∗∗*^	0.38 (0.12–1.26)	0.45 (0.18–1.12)	1.07 (0.34–3.35)	0.78 (0.3–2.04)	1.35 (0.62–4.49)

Residence location						
Urban vs. rural	0.91 (0.49–1.69)	0.87 (0.43–1.74)	0.60 (0.31–1.14)	0.57 (0.27–1.20)	**0.48 (0.26**–**0.89)**^*∗*^	**0.41 (0.2**–**0.85)**^*∗*^

Personal history						
Absence vs. presence	0.50 (0.09–2.74)	0.43 (0.06–3.18)	0.60 (0.11–3.29)	0.86 (0.1–7.43)	1.34 (0.24–7.38)	1.35 (0.15–12.48)

Family history						
Absence vs. presence	1.23 (0.60–2.56)	1.13 (0.46–2.74)	1.30 (0.63–2.69)	1.01 (0.42–2.41)	1.43 (0.66–3.1)	0.94 (0.38–2.34)

^*∗*^*p* < 0.05; ^*∗∗*^*p* < 0.01; ^*∗∗∗*^*p* < 0.001.

**Table 5:** Practice questionnaires of skin cancer.

		Gender			
		Male		Female	
		(*n* = 212; 39.5%)		(*n* = 325; 60.5%)	
How often are you currently exposed to the sun?	Everyday	111	52.4	125	38.5
	Weekends	35	16.5	67	20.6
	3 to 5 days	66	31.1	133	40.9

How many hours are you exposed to the sun during the day?	<2 hours	75	35.4	151	46.5
	3 to 5 hours	72	34.0	140	43.1
	6 to 8 hours	48	22.6	27	8.3
	>9 hours	17	8.0	7	2.2

Do you have a sunscreen at home?	Yes	140	66.0	238	73.2
	No	72	34.0	87	26.8

Do you regularly use sunscreen?	Yes	118	55.7	221	68.0
	No	94	44.3	104	32.0

How often do you use the sunscreen?	Everyday	79	37.4	191	58.8
	Often (3 to 5 days)	72	33.8	71	21.8
	Only weekends or travels	61	28.8	63	19.3

How many times a day do you use it?	Once a day	161	75.7	224	68.8
	Twice a day	42	19.9	76	23.5
	Three times a day	9	4.4	25	7.7

What time of year do you use sunscreen?	Summer	102	47.9	133	40.9
	Winter	1	0.7	4	1.2
	All the year	109	51.4	188	57.9

What other sun protection measures do you use?	Hat	102	48.0	182	55.9
	Sunglasses	5	2.5	4	1.3
	Seek shade	4	2.0	9	2.9
	Wear clothing to cover the whole body	4	2.0	2	0.6
	Avoid the sun between 10 am and 4 pm	0	0.0	1	0.3
	None	31	14.5	44	13.4
	Multiple	66	31.0	83	25.6

Where do you get information about sun protection and skin cancer?	TV, radio, newspaper	144	67.7	224	68.9
	Social network	21	10.1	24	7.3
	Family, friends	19	8.9	30	9.1
	Prescription of a doctor	8	3.8	15	4.6
	Prescription of a dermatologist	4	1.9	18	5.5
	Health campaigns	9	4.4	10	3.2
	Others	7	3.2	4	1.4

## Data Availability

The data collected in this study do not have the ability to identify the participants; however, they are subject to a confidentiality agreement with the researchers. Anonymized information concerning the database can be shared upon reasonable request to the corresponding author by e-mail address: ksimbanarivera@gmail.com.

## Supplementary Materials

Supplementary Materials Supplementary materials include “KAP skin cancer questionnaire.”

## References

[1] Instituto Nacional del Cáncer (2015). *Estadísticas del Cáncer*.

[2] World Health Organization (2020). *Cáncer*.

[3] Roque Pérez L., González Escudero M. (2019). Radiación solar y percepción de riesgo sobre cáncer de piel, un tema para reflexionar. *Multimedia*.

[4] Cabrera Morales C. M., López-Nevot M. A. (2006). Efectos de la radiación ultravioleta (UV) en la inducción de mutaciones de p53 en tumores de piel. *Oncología*.

[5] Apalla Z., Lallas A., Sotiriou E., Lazaridou E., Ioannides D. (2017). Epidemiological trends in skin cancer. *Dermatology Practical & Conceptual*.

[6] Loza A., Simi M., Iribas J. (2011). Conocimiento sobre melanoma y prácticas de protección frente al sol en pacientes del Hospital Cullen de Santa Fe, Argentina. *Revista Argentina de Dermatología*.

[7] American Cancer Society (2020). *Ultraviolet (UV) Radiation*.

[8] Sordo C., Gutiérrez C. (2013). Cáncer de piel y radiación solar: experiencia peruana en la prevención y detección temprana del cáncer de piel y melanoma. *Revista Peruana de Medicina Experimental y Salud Pública*.

[9] Mercadillo Pérez P., Moreno López L. M. (2013). Fisiopatología del carcinoma epidermoide. *Dermatologia Revista Mexicana*.

[10] Castañeda Gamerosa P., Eljure Téllez J. (2016). El cáncer de piel, un problema actual. *Revista de la Facultad de Medicina UNAM*.

[11] SOLCA (2018). *Informe de Labores 2016–2018*.

[12] Lema Puruncaja C. E., Zuleta Mediavilla D. P. (2015). Solmáforo (Semáforo Solar): modelo ambiental de alerta por exposición a la radiación solar en Quito.

[13] Carlos-Ortega B., García-Hidalgo L., Juárez-Navarrete L. (2020). Variables de conocimientos, actitudes y hábitos en sujetos mexicanos que acuden a jornadas de detección de cáncer de piel. *Dermatología Revista Mexicana*.

[14] Coca N. A. G., Rincón E. H. H., Ruíz J. C. (2016). El impacto de la prevención primaria y secundaria en la disminución del cáncer de piel. *CES Salud Pública*.

[15] de Ey Censos I. N. (2020). *Proyecciones poblacionales*.

[16] AlGhamdi K. M., AlAklabi A. S., AlQahtani A. Z. (2016). Knowledge, attitudes and practices of the general public toward sun exposure and protection: A national survey in Saudi Arabia. *Saudi Pharmaceutical Journal*.

[17] Geller A. C., Greinert R., Sinclair C. (2010). A nationwide population-based skin cancer screening in Germany: proceedings of the first meeting of the international task force on skin cancer screening and prevention (September 24 and 25, 2009). *Cancer Epidemiology*.

[18] El Rhazi K., Bennani B., El Fakir S. (2014). Public awareness of cancer risk factors in the Moroccan population: a population-based cross-sectional study. *BMC Cancer*.

[19] Wan M., Hu R., Li Y. (2016). Attitudes, beliefs, and measures taken by parents to protect their children from the sun in Guangzhou city, China. *Photochemistry and Photobiology*.

[20] Yan S., Xu F., Yang C. (2015). Demographic differences in sun protection beliefs and behavior: A community-based study in Shanghai, China. *International Journal of Environmental Research and Public Health*.

[21] Mufti S. T. (2012). Pattern of cutaneous melanoma at king AbdulAziz university hospital, Jeddah, Saudi Arabia. *Pakistan Journal of Medical Sciences*.

[22] Saridi M., Lionis S., Toska A. (2016). Evaluation of students’ knowledge and attitudes on sun radiation protection.

[23] Almuqati R. R., Alamri A. S., Almuqati N. R. (2019). Knowledge, attitude, and practices toward sun exposure and use of sun protection among non-medical, female, university students in Saudi Arabia: a cross-sectional study. *International Journal of Women’s Dermatology*.

[24] Spradlin K., Bass M., Hyman W., Keathley R. (2010). Skin cancer: knowledge, behaviors, and attitudes of college students. *Southern Medical Journal*.

[25] Cheng S., Lian S., Hao Y. (2010). Sun-exposure knowledge and protection behavior in a north chinese population: a questionnaire-based study. *Photodermatology, Photoimmunology & Photomedicine*.

[26] Skin Cancer Facts & Statistics (2020). The skin cancer foundation. https://www.skincancer.org/skin-cancer-information/skin-cancer-facts/.

[27] Urasaki M. B. M., Murad M. M., Silva M. T., Maekawa T. A., Zonta G. M. A. (2016). Práticas de exposição e proteção solar de jovens universitários. *Revista Brasileira de Enfermagem*.

[28] Carlos-Ortega B., García-Hidalgo L., Juárez-Navarrete L. (2019). Variables de conocimientos, actitudes y hábitos en sujetos mexicanos que acuden a jornadas de detección de cáncer de piel. *Dermatología Revista Mexicana*.

