# Supplementary material for: Knowledge, Attitude, and Practice (KAP) Survey toward Skin Cancer among Ecuadorian Population
Source: Dermatol Res Pract. 2021 Aug 4;2021:5539149. doi: 10.1155/2021/5539149 (PMC8357523; doi:10.1155/2021/5539149)
Supplement: Supplementary Materials — include “KAP skin cancer questionnaire.” [file 5539149.f1.zip › 5539149.f1/Questionnaire available of KAP Skin cancer - Previous request to corresponding author .pdf]

Deje en blanco

➲ [ ]

Conocimientos, actitudes y prácticas sobre la exposición a radiación solar y su asociación con el desarrollo de carcinoma basocelular

P1. **Provincia de realización de encuesta**

Por favor introduzca

P2. **Fecha de la entrevista**

Por favor introduzca una fecha válida

Dia	Mes	Año

I Factores Demográficos

Marque con una X la respuesta dada por el entrevistado, no le induzca la respuesta.

P3. **Sexo**

Seleccione sólo una opción

- Masculino ○ 1
- Femenino ○ 2

P4. **Si P3 es del sexo femenino, responder si está embarazada**

Seleccione sólo una opción

- Si ○ 1
- No ○ 2

P5. **Edad**

Coloque número y Seleccione sólo una opción

- < 20 años ☐ 1
- 21-30 años ☐ 2
- 31-40 años ☐ 3
- 41-50 años ☐ 4
- 51-60 años ☐ 5
- 61-70 años ☐ 6
- 71-80 años ☐ 7
- > 81 años ☐ 8

P6. **Raza**

Seleccione sólo una opción

- Mestizo ☐ 1
- Montubio ☐ 2
- Afroecuatoriano ☐ 3
- Indígena ☐ 4
- Blancos ☐ 5
- Otros ☐ 6

P7. **Estado civil**

Seleccione sólo una opción

- Soltero ☐ 1
- Casado ☐ 2
- Unión libre ☐ 3
- Separado ☐ 4
- Divorciado ☐ 5
- Viudo ☐ 6

P8. **Lugar de nacimiento**

Por favor introduzca una provincia y cantón

P9. **Lugar de residencia**

Por favor introduzca una provincia y cantón

P10. **¿Cuánto tiempo en años, usted vive en el lugar de la residencia?**

Seleccione sólo una opción

- 0-10 ☐ 1
- 11-20 ☐ 2
- 21-30 ☐ 3
- 31-40 ☐ 4
- 41-50 ☐ 5
- 51-60 ☐ 6
- 61-70 ☐ 7
- >70 años ☐ 8

II Factores Socioeconómicos y culturales

Marque con una X la respuesta dada por el entrevistado, no le induzca la respuesta.

P11. **¿Cuánta es el ingreso conjunto de la familia?**

Seleccione sólo una opción

- < $386 ☐ 1
- $386-$650 ☐ 2
- $650-1000 ☐ 3
- > $1000 ☐ 5

P12. **¿Cuántas personas viven en su casa?**

Seleccione sólo una opción

- <2 personas ☐ 1
- 2-4 personas ☐ 2
- 4-6 personas ☐ 3
- >6 personas ☐ 4

P13. **¿Hasta que año usted estudió?**

A continuación se muestra una tabla, señale con una X solo una respuesta, la obtenida del entrevistado.

		A		B	
1	Analfabeto	SI	[ ]	NO	[ ]
2	Preescolar	SI	[ ]	NO	[ ]
3	Primaria (hasta 3er curso)	COMPLETA	[ ]	INCOMPLETA	[ ]
4	Secundaria (bachillerato)	COMPLETA	[ ]	INCOMPLETA	[ ]
5	Técnico	COMPLETA	[ ]	INCOMPLETA	[ ]
6	Superior 3er nivel	COMPLETA	[ ]	INCOMPLETA	[ ]
7	Superior 4to nivel	COMPLETA	[ ]	INCOMPLETA	[ ]

Respuestas Ejemplo si preescolar= 2A

P14. **¿Cuál es su ocupación?**

Seleccione sólo una opción

- Obreros (Albañil, Jardinero, Carpintero, Artesano) ☐ 1
- Ama de casa o empleada doméstica ☐ 2
- Agricultor ☐ 3
- Comerciante ☐ 4
- Estudiante ☐ 5
- Profesional ☐ 6
- Militar o Policía ☐ 7
- Jubilado ☐ 8
- Desempleado ☐ 9
- Mixto (más de una opción) ☐ 10

P15. **Lugar de trabajo**

Seleccione sólo una opción

- Oficina ☐ 1
- Exteriores ☐ 2
- Ambos ☐ 3
- No aplica ☐ 4

P16. **¿Ubicación del lugar de trabajo?**

Seleccione sólo una opción

- Urbano ☐ 1
- Suburbano ☐ 2
- Rural ☐ 3
- No aplica ☐ 4

P17. **¿Ubicación del sitio de residencia?**

- Urbano ☐ 1
- Suburbano ☐ 2
- Rural ☐ 3

P18. **¿Tiene agua potable?**

- Si ○ 1
- No ○ 2

P19. **¿Tiene alcantarrillado?**

- Si ○ 1
- No ○ 2

P20. **¿Tiene luz eléctrica?**

- Si ○ 1
- No ○ 2

III Factores clínicos

Marque con una X la respuesta dada por el entrevistado, no le induzca la respuesta.

P21. **Qué opción describe mejor su tipo de piel (según Fitzpatrick)?**

Seleccione sólo una opción

- Tipo I. Piel muy blanca, se quema fácilmente, siempre se pone rojo, no se pigmenta nunca, a la semana se despelleja. ☐ 1
- Tipo II Piel blanca, siempre se quema, se pigmenta ligeramente y se descama de forma notoria. ☐ 2
- Tipo III. Se quema moderadamente, se pigmenta facilmente en forma gradual y uniforme. ☐ 3
- Tipo IV. Casi no se pone rojo o se quema muy poco, siempre se pigmenta con facilidad. ☐ 4
- Tipo V. Rara vez se pone rojo, siempre se pigmenta con facilidad e intensidad (piel morena) ☐ 5
- Tipo VI. Nunca se quema, muy pigmentado (piel negra) ☐ 6

P22. **Antecende personal de cáncer de piel**

Seleccione sólo una opción

- Si ○ 1
- No ○ 2

P23. **Antecedente familiar de cáncer de piel**

Seleccione sólo una opción

- Si ○ 1
- No ○ 2

IV Conocimientos, actitudes y prácticas (CAP)

V Conocimiento de la enfermedad

Seleccione solo UNA RESPUESTA en las siguientes preguntas

P24. **¿Conoce usted los efectos perjudiciales o riesgos de la exposición solar?**

Seleccione sólo una opción

- Si ☐ 1
- No ☐ 2

P25. **Si la respuesta anterior es SI, responder¿Qué efectos negativos o perjudiciales conoce usted?**

Seleccione una o más opciones

- Quemadura solar ☐ 1
- Arrugas ☐ 2
- Envejecimiento ☐ 3
- Manchas ☐ 4
- Pecas ☐ 6
- Sequedad de piel ☐ 7
- Cáncer de piel ☐ 8
- Ninguno ☐ 9

P26. **¿Conoce usted sobre el cáncer de piel?**

Seleccione sólo una opción

- Si ☐ 1
- No ☐ 2

P27. ¿Sabe usted que existe una relación causal entre exposición solar y cáncer de piel?

- Si ☐ 1
- No ☐ 2

P28. ¿Sabe qué son los fotoprotectores?

- Si ☐ 1
- No ☐ 2

P29. Si P28 es SI, responder ¿Sabe para qué se utilizan los fotoprotectores?

- Si ☐ 1
- No ☐ 2

P30. ¿Conoce usted otras medidas de prevención del sol diferentes al fotoprotector solar?

- Si ☐ 1
- No ☐ 2

VI Actitud y práctica

Marque con una X la respuesta dada por el entrevistado, no le induzca la respuesta.

P31. Número de quemaduras solares que ha presentado alguna vez en la vida

Seleccione sólo una opción

- Nunca ☐ 1
- Una vez en la vida ☐ 2
- Dos veces en la vida ☐ 3
- Más de tres veces en la vida ☐ 4

P32. ¿Con qué frecuencia se exponía al sol durante su niñez y adolescencia?

Seleccione sólo una opción

- Todos los días ☐ 1
- Sólo fines de semana ☐ 2
- Ocasionalmente (3 a 5 días) ☐ 3

P33. ¿Con qué frecuencia se expone al sol actualmente?

Seleccione sólo una opción

- Todos los días ☐ 1
- Sólo fines de semana ☐ 2
- Ocasionalmente (3 a 5 días) ☐ 3

P34. ¿Cuántas horas aproximadamente se expone usted al sol durante el día?

Seleccione sólo una opción

- < 2 Horas ☐ 1
- 3 a 5 Horas ☐ 2
- 6 a 8 Horas ☐ 3
- > 9 Horas ☐ 4

P35. ¿Tiene en casa un fotoprotector solar?

Seleccione sólo una opción

- Si ☐ 1
- No ☐ 2

P36. ¿Regularmente usa fotoprotector?

Seleccione sólo una opción

- Si (siempre, a veces, rara vez) ☐ 1
- No ☐ 2

P37. Si P35 es SI; responder P37, P38, P39, P40. ¿Qué factor de protección (FPS) tiene el fotoprotector?

Seleccione sólo una opción

- 6 a 10 ☐ 1
- 15 a 25 ☐ 2
- 30 a 50 ☐ 3
- > 50 ☐ 4
- No sabe ☐ 5

P38. ¿Cuál fue el costo aproximado?

Seleccione sólo una opción

- Menor a $10 ☐ 1
- De $11 a $20 ☐ 2
- De $21 a $30 ☐ 3
- Mayor a $31 ☐ 4
- No sabe ☐ 5

P39. ¿Considera que el valor es accesible?

Seleccione sólo una opción

- Si ☐ 1
- No ☐ 2

P40. ¿Dónde adquirió el fotoprotector?

Seleccione sólo una opción

- Tiendas o Markets ☐ 1
- Farmacias ☐ 2
- Revistas por catálogo ☐ 3
- Otros ☐ 4

P41. Si P36 es SI; Responder P41,P42,P43 ¿Con qué frecuencia usa el fotoprotector?

Seleccione sólo una opción

- Todos los días ☐ 1
- Ocasionalmente (3 a 5 días) ☐ 2
- Solo en salidas y fines de semana ☐ 3

P42. ¿Cuántas veces al día lo usa?

Seleccione sólo una opción

- Una vez al día ☐ 1
- Dos veces al día ☐ 2
- Tres veces al día ☐ 3

P43. **¿En qué época del año usa fotoprotector solar?**

Seleccione sólo una opción

- Verano (Junio a Noviembre) ☐ 1
- Invierno (Diciembre a Mayo) ☐ 2
- Todo el año ☐ 3

P44. **Si P36 es NO; Responder P44 ¿Cuáles fueron sus razones para no usarlo?**

Seleccione una o más opciones

- Porque son muy costosos ☐ 1
- Porque no tiene tiempo ☐ 2
- Se olvida de colocarse ☐ 3
- Porque no le gusta la sensación ☐ 4
- Porque quiere broncearse ☐ 5
- Piensa que no es necesario para su tipo de piel ☐ 6
- Piensa que no es necesario todo el año ☐ 7
- Otros (Especificar) ☐ 8

P45. **¿Usa usted alguna otra medida de prevención diferente al fotoprotector solar?**

Seleccione sólo una opción

- Si ☐ 1
- No ☐ 2

P46. **¿Qué otras medidas de protección solar usa?**

Seleccione una o más opciones

- Uso de sombrero o gorra ☐ 1
- Uso de lentes de sol ☐ 2
- Busca la sombra ☐ 3
- Usa ropa que cubra la mayor cantidad de piel ☐ 4
- Evitar el sol entre las 10am y las 4pm ☐ 5
- Ninguna ☐ 6

P47. **¿De dónde obtiene información sobre fotoprotección y cáncer de piel?**

Seleccione una o más opciones

- Medios de comunicación (TV, radio, periódico) ☐ 1
- Redes sociales ☐ 2
- Familia, amigos ☐ 3
- Prescripción Médico General ☐ 4
- Prescripción Médico Dermatólogo ☐ 5
- Campañas de salud ☐ 6
- Otros (Especificar) ☐ 7

P48. **¿Cree usted que las personas lucen más saludables con un bronceado?**

- Si ☐ 1
- No ☐ 2

P49. **¿Cuándo usted va a la playa o a un día de campo. ¿Busca broncearse intencionalmente?**

- Si ☐ 1
- No ☐ 2

P50. **En días no soleados, nublados, es necesario el uso de protector solar.**

- Si ☐ 1
- No ☐ 2

Teléfono

ENTREVISTADOR NOMBRE Y FIRMA
