# Supplementary material for: Knowledge, Attitude, and Practice (KAP) Survey toward Skin Cancer among Ecuadorian Population
Source: Dermatol Res Pract. 2021 Aug 4;2021:5539149. doi: 10.1155/2021/5539149 (PMC8357523; doi:10.1155/2021/5539149)
Supplement: Supplementary Materials — include “KAP skin cancer questionnaire.” [file 5539149.f1.zip › 5539149.f1/supplementary description.docx]

Supplementary description:

“Database available of KAP Skin cancer”

“KAP skin cancer questionnaire”
